# Lifetime Distributions from Tracking Individual BC3H1 Cells Subjected to Yessotoxin

**DOI:** 10.3389/fbioe.2015.00166

**Published:** 2015-10-21

**Authors:** Mónica Suárez Korsnes, Reinert Korsnes

**Affiliations:** ^1^Department of Chemistry, Biotechnology and Food Science, Norwegian University of Life Sciences, Ås, Norway; ^2^Norwegian Institute of Bioeconomy Research, Ås, Norway; ^3^Norwegian Defense Research Establishment, Kjeller, Norway

**Keywords:** cell tracking, lifetime statistics, yessotoxin, cell death, inter-cellular influence

## Abstract

This work shows examples of lifetime distributions for individual BC3H1 cells after start of exposure to the marine toxin yessotoxin (YTX) in an experimental dish. The present tracking of many single cells from time-lapse microscopy data demonstrates the complexity in individual cell fate and which can be masked in aggregate properties. This contribution also demonstrates the general practicality of cell tracking. It can serve as a conceptually simple and non-intrusive method for high throughput early analysis of cytotoxic effects to assess early and late time points relevant for further analyzes or to assess for variability and sub-populations of interest. The present examples of lifetime distributions seem partly to reflect different cell death modalities. Differences between cell lifetime distributions derived from populations in different experimental dishes can potentially provide measures of inter-cellular influence. Such outcomes may help to understand tumor-cell resistance to drug therapy and to predict the probability of metastasis.

## 1 Introduction

This work calls for attention to describe variability of individual cell responses in clonal cell populations subject to toxic exposure. The present work evaluates individual BC3H1 cell responses after toxic exposure to the marine toxin yessotoxin (YTX). YTX is a small molecule compound, which can trigger a broad spectrum of cellular responses for possible medical applications (Korsnes et al., 2006a,b, 2014; López et al., 2008, 2011a,b; Korsnes, 2012; Alonso et al., 2013). Time-lapse observations of BC3H1 cells exposed to YTX provide a description of the diversity of individual cell responses to toxic exposure. A small fraction of cells withstand the exposure much more than others, whereas some cells die long before the majority. The presence of such minorities may have interest for assessments of long term effects of a toxin. Parameters in simulation models of cellular responses to toxic insults may be tuned to reproduce the complexity and features of observed lifetime distributions. Tracking of individual cells can in this way contribute to reverse engineering of cellular signaling.

Typical cell viability analyzes are based on measurements of cell metabolism at a limited set of time points. Some of these measurements are intrusive (i.e., affecting the target of measurement). Subsets of dead cells may tend to dissolve in the growth media and in this way being excluded from temporally sparse measurements. Flow cytometry is used due to its massive throughput combined with fluorescent labeling (Nolan and Sklar, 1998). However, it also samples only at specific time points and do not follow single cells over time. Wei et al. (2008) demonstrated non-invasive detection of cell viability based on wavelet decomposition of dark field microscopy images of cells. However, no current cell viability analysis directly provides measurements of single cell lifetime distributions. The inherent uncertainties of common measurements of cell viability may partly explain the lack of attention to the stochastic aspects of single cell behavior.

Lifetime distributions of cells after toxic exposure may affect the understanding of results from bulk cell analyzes. Such techniques do not provide the correct distribution of a response, which is important to develop mathematical descriptions of cellular behavior (Teruel and Meyer, 2002). Cell viability assays, for example, typically provide estimates of aggregate properties of many cells subject to a common treatment. An example of such an aggregate property is the widely used half maximal inhibitory concentration (IC_50_). This measure normally results from exposing cell populations to a compound at different concentrations for estimating which concentration is needed to inhibit given biological processes by half. It is tempting to attribute variations of results form such assays to procedural errors or unavoidable noise (Xia et al., 2014). The next step in following this intuition is to reduce uncertainty by repeating experiments and to calculate the average of the results. This value is assumed to converge to a biologically meaningful quantity with increased number of experiments. However, the parameter IC_50_ may not reflect biological properties of any individual cell. The present lifetime distributions also indicate that IC_50_ can be sensitive to time points for the cell viability measurements which it often relies on. Hence, one may expect it to be reproducible only if it is defined in terms of experimental conditions in addition to its original definition.

An experiment can, from a practical point of view, be considered deterministic only if the variation of outcomes decreases with decreased perturbations in experimental conditions (at least when they are small enough). The opposite of deterministic is normally phrased as stochastic or chaotic. Throwing dice can, therefore in practice, only be described probabilistic. Note that the outcome from dice throwing has no direct physical meaningful expectation value (3.5). There is similarly no guarantee that the mean value of results from cell assays has a direct biological interpretation since the result may be considered stochastic and multi-modal with unlikely outcomes “between” different modes. The average of values from measurements of single cells in a cell assay may also not reflect the average over several experiments.

Several authors address variability among individual cells in clonal populations. Sources of the variability are the proximity to an inductive signal from a neighboring cell, their lineage, oncogenic lesions, natural differences in protein levels, cell-cycle state and epigenetic differences (Rubin, 1990; Elowitz et al., 2002; Rieder and Maiato, 2004; Weaver and Cleveland, 2005; Losick and Desplan, 2008; Huang, 2009; Spencer and Sorger, 2011; Fromion et al., 2013). Although, interline and intraline variation is expected in cancer cell populations, cell fate decisions do not appear to be genetically predetermined, because sister cells can undergo different cell fates (Gascoigne and Taylor, 2008). Variability may also result from noise in gene expression (Elowitz et al., 2002; Losick and Desplan, 2008; Raychaudhuri et al., 2008; Spencer et al., 2009).

Stochasticity requires both a means to generate noise but at the same time mechanisms to stabilize decisions reached in response to it. Noise alone is insufficient to create binary switches between alternative cell fates and therefore mechanisms to amplify fluctuations are necessary to stabilize one choice or another (Losick and Desplan, 2008). Stochastic choices can make cells autonomous and cell fate decisions may be independent of other nearby cells.

If cells affect each other during an experiment, the assay may not be “ergodic” due to collective effects. Ergodicity is a central term in mathematical statistics and it is often implicitly assumed in many experimental settings. The assumption can mean that the average over individuals in a cell population due to a bulk treatment reflects the average over many cell assays. Interactions between cells may make this assumption unrealistic and can in principle make averages of measures from different assays not directly meaningful. It is well known that simple ways of interactions between individuals can lead to complex collective behavior in higher organisms (for example flocking). There should be no reason to assume “simpler” statistics for cells.

A major challenge is to identify what aspects of cellular variability bear significant biological meaning (Li and You, 2013). A variety of sources can induce diversity, which may presumably not be critical for its biological role. Variability may stem from redundant perturbations and a source of randomization can create variation in different mechanisms. It is unclear, however, to what extent and under what situations cellular mechanisms are used to exploit gene expression variability that can affect cell phenotype (Blake et al., 2006). Research on the yeast *S. cerevisiae* reveals that increased variability in gene expression can provide an evolutionary advantage. Blake et al. (2003) and Becskei et al. (2005) suggested that variation in the rates of transition between different states of promoter activity in the TATA box may play a role in determining the level of stochasticity in gene expression. The sequence of the TATA box can, therefore, enable cell–cell variability in gene expression being beneficial after an acute change in environmental conditions (Blake et al., 2006).

This work demonstrates that cell tracking can provide information on cellular variability. Tracking many objects in changing environments has in general many applications and work on it has a long history over 50 years and now entering also biomedical research (Mallick et al., 2013). Cell tracking is an emerging technology based on treatment of cells (labeling and contrast enhancements), various imaging techniques (microscopy) and also algorithms for automatic feature extraction. The initiative *Open Bio Image Alliance*^1^ reflects this development organizing competitions on cell tracking (so-called “challenges”) to promote development of open-source software for cell tracking. Sacan et al. (2008) represents an early contribution in this development. Note that cell feature extraction from images can help to resolve ambiguities during multi-target tracking. The capacity to distinguish between individual cells is, therefore, relevant to relax requirements on data collection or to increase reliability. Holmquist et al. (1978) represents an early attempt to formalize automatic feature extraction. There are numerous similar later attempts, which may be relevant for joint tracking and classifying of many cells (Mattie et al., 2000; Wei et al., 2008; Basu et al., 2014; Jusman et al., 2014), and which can serve to apply theory of joint multi-target classification and tracking (Mahler, 1994; Goodman et al., 2013).

The present work illustrates possible information gain from computer-assisted tracking of individual BC3H1 cells after exposure to YTX. The actual tracking was based on visual control via computer terminal to produce reliable lifetime data. A realistic way to develop systems for quick and low cost estimation of individual cell lifetimes is first to develop a usable “hybrid” approach where automatic algorithms gradually replace visually based control. This strategy allows early to produce lifetime statistics without bias due to automatic algorithms tending to lose tracks of, for example, specially long living or motile cells.

Following many cells over time can promote awareness of sub-populations and other types of diversity not otherwise easily detected. It can potentially complement measurements of the proteomic dynamics and gene sequencing in individual cells and contribute to clinical assessments. Results from such tracking may also help to reverse engineer cellular processes to develop simulation models for better prediction of how toxins may affect organisms.

## 2 Yessotoxin

Yessotoxin (YTX) is a small molecule marine polyether compound produced by dinoflagellates and which can accumulate in filter-feeding bivalves (Murata et al., 1987; Ogino et al., 1997; Satake et al., 1997; Draisci et al., 1999; Paz et al., 2004). It has numerous analogs (Miles et al., 2005). The understanding of its mechanisms of action in cells is developing (Malaguti et al., 2002; Alfonso et al., 2003; Malagoli et al., 2006; Korsnes et al., 2007, 2013; Martín-López et al., 2012; Fernández-Araujo et al., 2014, 2015; Rubiolo et al., 2014). It can at low concentrations induce various cytotoxic effects and programed cell death mechanisms in different types of cells (Leira et al., 2002; Ronzitti and Rossini, 2008; Young et al., 2009; Korsnes and Espenes, 2011; Korsnes et al., 2011). The diversity of toxic responses raises attention for potential medical and therapeutic applications of YTX (López et al., 2008, 2011b; Korsnes, 2012; Alonso et al., 2013; Alonso and Rubiolo, 2015; Fernández-Araujo et al., 2015).

The specific molecular target of YTX is generally unclear. However, phosphodiesterases, heterogeneous nuclear ribonucleoproteins (hnRPps), heat shock, and Ras proteins have been reported as YTX targets in human lymphocytes, HepG2 cells, and blood cell membranes (Alfonso et al., 2003; Young et al., 2009; Ujihara et al., 2010). The specific organelle targets appear to be the mitochondria and the ribosome (Bianchi et al., 2004; Korsnes et al., 2006a, 2014).

## 3 Materials and Methods

### 3.1 Toxin

YTX was provided by Christopher. O. Miles at the National Veterinary Institute of Norway. YTX was dissolved in methanol as a 50-μM stock solution. The stock solution was diluted in Dulbecco’s modified Eagle’s medium (DMEM, Sigma) achieving a final concentration of 100 nM YTX and 200 nM in 0.2% methanol. Control cells were incubated with 0.2% methanol as vehicle. Control cells and treated cells were exposed up to 48 h.

### 3.2 Cell Culture

BC3H1 cell lines were isolated from primary cultures derived from mouse (ATCC Number CRL-1443). BC3H1 cells closely resemble cells in an arrested state of skeletal muscle differentiation than smooth muscle cells (Schubert et al., 1974; Taubman et al., 1989). Both cell lines were purchased from the American Type Culture Collection (Manassas, VA, USA) at a seeding density of 2 × 10^6^ cells per cm^2^. Cells were maintained undifferentiated at 37°C in a humidified 5% CO_2_ atmosphere.

### 3.3 Time-Lapse Video Microscopy

BC3H1 cells were plated in 35 mm × 10 mm glass bottom dishes (Willco, USA) for time-lapse imaging. Cells were cultured in medium (DMEM with phenol red, containing 50 nM Hepes, 7.2pH, and 20% fetal bovine serum). The temperature in the box was maintained at 37°C using a heat controller. Cells were observed in a Zeiss LSM 700 microscope and analyzed using the phase contrast optics. All images were taken using a Plan Apochromat 20×/0.8 ph2M27 objective. The time-lapse images were generated using a ZEN 2010 imaging software. Cells were continuously imaged each 2.5 min for up to 48 h by time-lapse microscopy using phase contrast optics. Tracking of randomly selected cells through these sub-sequential images provided direct samples of lifetimes of cells.

### 3.4 Cell Tracking

The time-lapse video microscope provided images in jpeg format for each 2.5 min. The co-author (Reinert Korsnes) developed a computer program to support cell tracking through these images. It was written in Ada 2012 using for graphics the GLOBE_3D system developed by Gautier de Montmollin.^2^ The Ada compiler was GNAT Ada from AdaCore.^3^ A common type laptop computer running Linux was used for processing.

**Figure 1:**
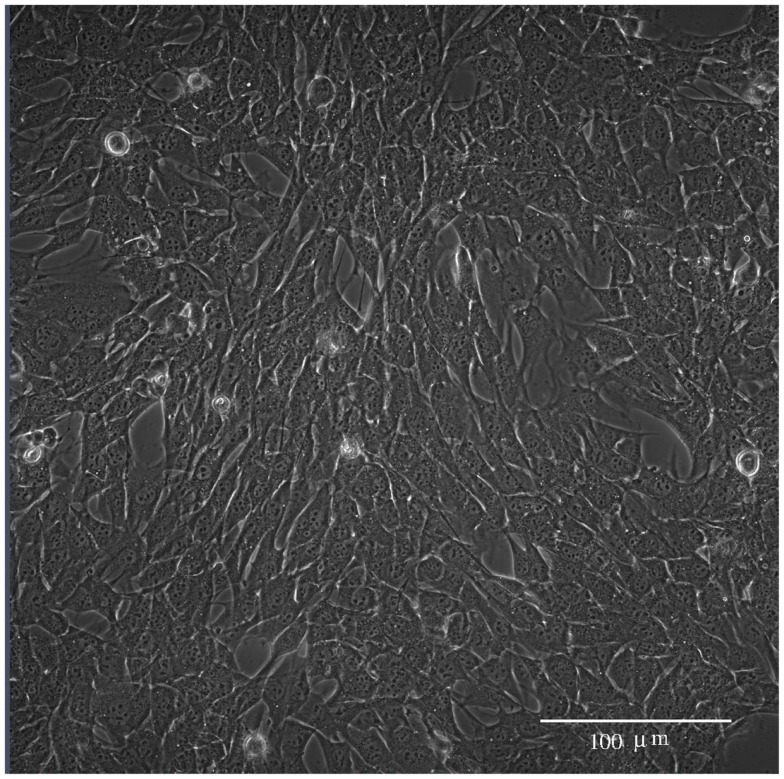
**Phase contrast image of BC3H1 cells short after YTX exposure**.

**Figure 2:**
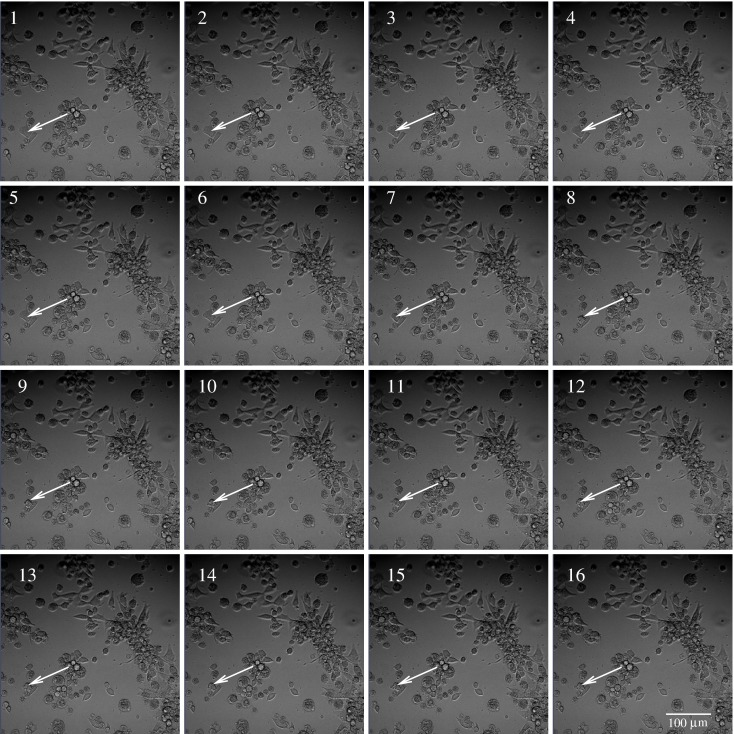
**Example of subsequent images of BC3H1 cells exposed to 100 nM YTX**. Images taken at 2.5 min interval. The time of image 1 is 30 h treatment after exposure to YTX. The white arrow illustrates tracking of a cell, which dies during the time of image 6–9.

The present cell tracking is computer-aided and not fully automatic. The computer program, which we developed, kept track of a user controlled cursor pointing on a cell in a video on a desktop screen (this design makes it ready to include automation support). This video did consist of displaying images originally in jpeg format from the microscope. No special image treatments were applied. The user could affect the display of the image sequence (enlargement, speed, and direction in time). Figures 1 and 2 illustrate the imagery data. The computer program did for each track generate a random position more than 100 μm from the image border. The nearest cell inside this inner zone of the image at start of recording, then, was chosen for tracking. The reason to choose (by random) cells away from the image border was to avoid that they moved outside the image scene before dying. 150 cells were tracked in each experimental well. Few of these cells (less than ten) did divide during tracking. A track followed by random one of the daughter cells, when it encountered a cell division. No tracks were lost.

The lifetime of a cell was defined as the duration from start of toxin exposure to when it died. Cells in the image scene normally moved and changed shape at least each 10–20 min. A significant part of the cells did undergo a necrosis-like death rounding up to a steady shape. When finding a cell reached this steady state, the cell track was followed back in time to detect the time of start of this steady state. This time was defined as time of death. The present estimate of this time was within a precision of less than 30 min. Tracked cells undergoing apoptosis-like death in the way that they fractionated during few minutes, were given time of death at the moment of this event. The above procedure is relatively simple to implement as a computer algorithm.

### 3.5 Kernel Density Estimation

Let the stochastic variable *T* represent lifetime of a randomly selected cell after being exposed to a toxin. The kernel density estimation (KDE) provides a non-parametric way to reconstruct the probability density of *T* from random samples (Rosenblatt, 1956; Parzen, 1962). Let *t*_1_, *t*_2_, … , *t_n_* represent such samples (measurements) of lifetimes for *n* randomly selected cells. Assume a distribution *m* (probability measure) equally concentrated on the points *t*_1_, *t*_2_, … , *t_n_* of the real line such that

(1) $$m\left(\left\{t\right\}\right)=\left\{\begin{array}{cc} \frac{1}{n}\; & \text{if}\;t\in \left\{t_{1},t_{2},\ldots ,t_{n}\right\}\\ 0\; & \text{otherwise} \end{array}\right.$$

Given the Gaussian kernel:

(2) $$K\left(t\right)=\frac{1}{\sqrt{2\pi }}e^{-\frac{t^{2}}{2}}$$

which for *h* > 0 gives a family of kernels $K_{h}(t)=\frac{1}{h}K\left(\frac{t}{h}\right)$ conserving its integral $\left(\int K_{h}\left(x\right)\;dx=1\right)$. The parameter *t* here represents time and *h* is termed bandwidth. The convolution between the discrete (singular) measure *m* and a kernel *K_h_* gives a “smooth version” *p_h_* of the distribution *m*:

(3) $$p_{h}\left(t\right)=\left(K_{h}*m\right)\left(t\right)=\int_{R}K_{h}\left(t-s\right)\mathrm{dm}\left(s\right)$$

This smooth (“diffused”) version of the singular measure *m* is considered as an estimate of the distribution of the original stochastic variable *T* above. The present work applies kernel density estimation on the above simple level justified by the principle of Occam’s razor. Note, however the similarities of the above convolution [Eq. (3)] and diffusion (for example physical heat conduction) provide inspiration for more precise estimation (Botev et al., 2010; Berry and Harlim, in press).

### 3.6 Weibull Analysis

The Weibull distribution is known as “Type 3” of three possible types of approximate distributions of the extreme (maximum or minimum) of a set of random variables (Fisher and Tippett, 1928; Leadbetter et al., 1983). It covers the case where the extreme value has a light tail with finite upper bound. It is a versatile and widely used model for lifetimes of successful functioning of systems in general. Its applicability is so wide that lifetime (or failure) analysis has been termed “Weibull analysis.” A convex combination of two Weibull distributions can express the distribution of life length of systems of two possible (but unknown) types.

A single population two parameter Weibull probability density distribution has the following form:

(4) $$f\left(t;\lambda ,k\right)=\left\{\begin{array}{cc} \frac{k}{\lambda }{\left(\frac{t}{\lambda }\right)}^{k-1}e^{-{\left(\frac{t}{\lambda }\right)}^{k}}\; & \text{if}\;t\geq 0\\ 0\; & \text{otherwise} \end{array}\right.$$

where *k* is a shape parameter and λ here defines time scale. The corresponding cumulative distribution is

(5) $$F\left(t;\lambda ,k\right)=1-e^{-{\left(\frac{x}{\lambda }\right)}^{k}}$$

Assume the convex combination of two Weibull distributions:

(6) $$f\left(t\right)=\omega_{1}f\left(t;\lambda_{1},k_{1}\right)+\omega_{2}f\left(t;\lambda_{2},k_{2}\right)$$

where ω_1_ + ω_2_ = 1 and ω_*i*_ ≥ 0. *f* (*t*) is also a probability density function (non-negative and with integral equal to 1). The corresponding cumulative distribution is

(7) $$F\left(t\right)=\omega_{1}F\left(t;\lambda_{1},k_{1}\right)+\omega_{2}F\left(t;\lambda_{2},k_{2}\right)$$

## 4 Results

**Figure 3:**
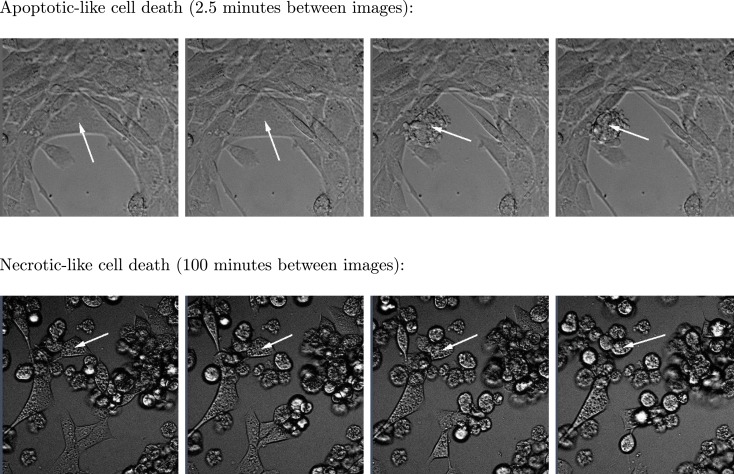
**Two sequences of four images respectively showing typical apoptotic- and necrotic-like death events among BC3H1 cells exposed to yessotoxin**. The necrotic-like cell death process is much slower than the apoptotic-like cell death.

**Figure 4:**
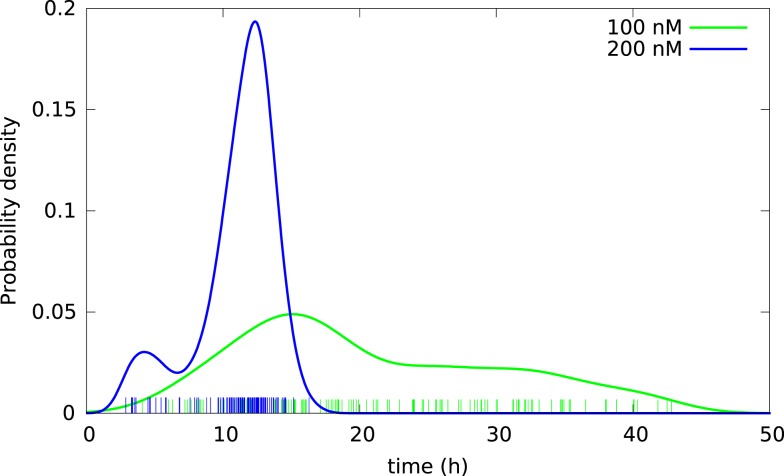
**Kernel density estimates of distributions of lifetimes of BC3H1 cells after YTX exposure at concentrations 100 and 200 nM**. Vertical bars indicate individual observations (samples).

Subsequent phase contrast microscopy images in this work provide lifetime distributions for BC3H1 cells after YTX exposure. Figure 1 shows an example of such an image at start of exposure, whereas Figure 2 shows subsequent similar images after 30 h of exposure. The cells obviously do not behave uniformly. Some of them exhibit apoptotic-like cell death morphologies, whereas others appear to die necrotic-like (Figure 3). Figure 4 shows kernel density estimates [cf Eq. (3)] of distributions for observed lifetimes of BC3H1 cells after the start of exposure to YTX at concentrations of 100 and 200 nM. The bandwidth *h* is, here, according to Silverman’s rule of thumb (Silverman, 1986; Bowman and Azzalini, 1997). The distribution for 100 nM has a significant upper tail indicating a mixture of mechanisms in action when the cells die. A single peak seems to dominate the distribution for 200 nM.

**Figure 5:**
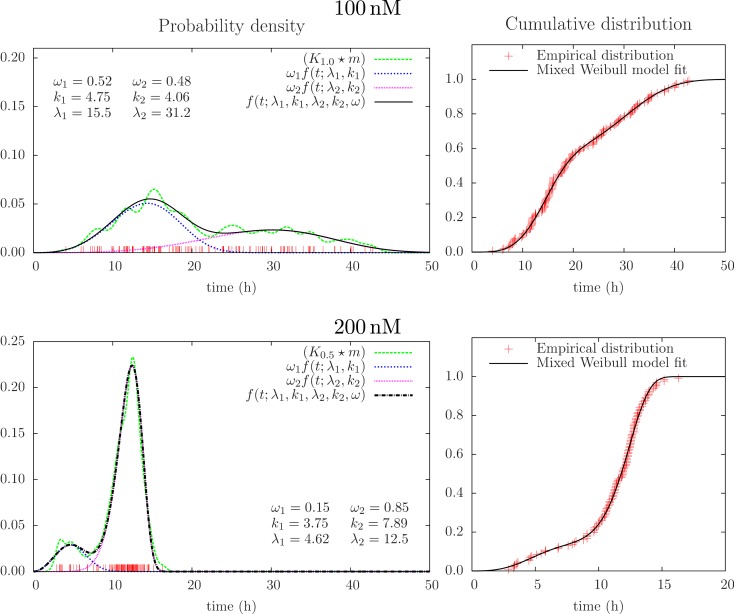
**Results from mixed Weibull analysis giving estimates of distributions of lifetimes of BC3H1 cells after YTX exposure at concentrations 100 and 200 nM**. The parameters for the mixed Weibull distribution (*k* and λ) result from model fit to the empirical cumulative distribution (right). Red vertical bars indicate individual observations (samples). The smoothed histograms *K*_1.0_ × *m* and *K*_0.5_ × *m* [cf Eq. (3)] are here for visual control and illustration.

Successful parametric ways to reconstruct probability distributions from measurements typically require fewer samples as compared to non-parametric ways, or it can provide more precise results given the same data. This is intuitively reasonable since the approach exploits restrictions on the set of possible outcomes from experiments and in this simple way represents sparse sampling or compressive sensing. Optimal use of data is here of interest in possible applications of cell tracking since tracking may cost and early information on lifetime distributions (over many days) may have direct interest in clinical situations (for example to monitor and control development of cancer). Parametric reconstruction can also support understanding of underlying processes. Figure 5 shows an attempt to fit a mixed (bimodal) Weibull model [Eq. (7)] to the same lifetime data, as in Figure 4. It shows the result from fitting this model to the empirical distribution function (varying the parameters: *k*_1_, λ_1_,*k*_2_, λ_2_, ω_*i*_ condition on ω_1_ + ω_2_ = 1, ω_*i*_ ≥ 0). Figure 5 also illustrates the probability density function for these parameters [cf Eqs (4) and (6)].

**Figure 6:**
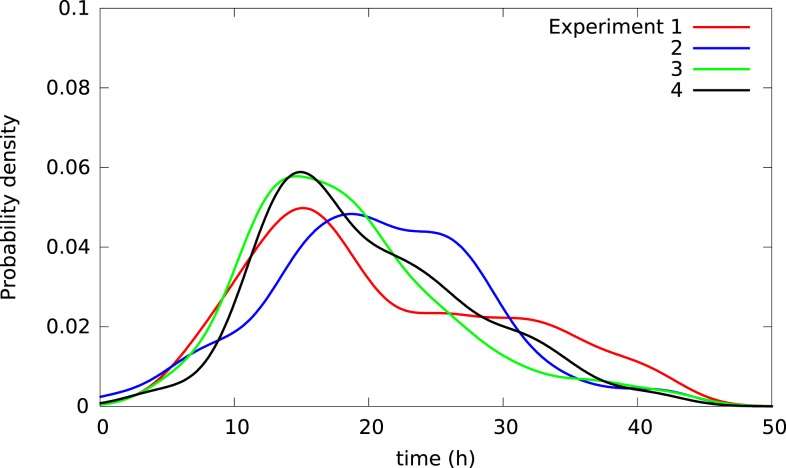
**Kernel density estimates of distributions of lifetimes in four separate populations of BC3H1 cells after exposed to 100 nM YTX (i.e., each of the four populations were in separate experimental wells)**. Influence between cells in the same well may explain the difference between the distributions. These variations between distributions can, therefore, potentially provide measures of such inter-cellular influence.

Cells may affect each other in experimental wells via, for example, cytoskeletal contacts and in ways affecting survival after toxic exposure. Hence, the lifetimes of cells in the same experimental well may not be independent giving somehow different lifetime distributions for cells in distinct wells. Figure 6 illustrates this possible effect showing lifetime distributions of BC3H1 cells exposed to 100 nM YTX in four different populations (experiments). The distributions significantly vary despite carefulness to repeat the experiments the same way.

## 5 Discussion

The present experiments show examples on how cells in the same cell line can display individual variation in their response to a toxic exposure. Korsnes (2012) showed still photos illustrating diversity in cell death response after YTX exposure. Sub-sequential images provide more reliable interpretations since it gives information from before and after given time points. Tracking from time lapse of living cells may therefore, when the appropriate instrumentation and software tools become more available, be a valuable tool to estimate how cells react to stress. Clarification of the biological significance of the present observed variations, however, needs further investigations. For example, Figure 5 indicates two dominating mechanisms of cell death, but lifetime data from similar experiments do not exhibit the same structure.

Differences in cell fate decisions in isogenic populations have been explained by oncogenic lesions, which are genetically predetermined, cell-cycle state or as a result of their lineage or their proximity to an inductive signal from other cells (Rieder and Maiato, 2004; Weaver and Cleveland, 2005). However, stochastic distributions of cell fates can also take place independent of cell cycle or history (Losick and Desplan, 2008; Spencer et al., 2009).

Different mechanisms for cellular variability may be biologically significant and therefore evolutionary conserved. Examples of such mechanisms in multi-cellular organisms driving specific cell fates where stochastic activation is coupled in some cases with a positive feedback loop or in other cases with a negative feedback regulation, have been described (Heitzler and Simpson, 1991; Serizawa et al., 2003; Lomvardas et al., 2006).

Mechanisms that have evolved to exploit stochastic variation at the level of single cells or whole tissues during development may also operate at much higher levels of biological organization, such as insect colonial organisms (Hölldobler and Wilson, 2008). Stochastic establishment of gamer-gates may be favored by natural selection to ensure the emergence of reproductive individuals upon the demise of the queen. When the queen or other gamer-gates die, the colony must maximize its potential for replacing these reproductively dominant individuals, which may otherwise be limited by deterministic rules (Kilfoil et al., 2009).

It may be reasonable to speculate that randomization in cell populations can have a function in contexts of optimization. It is a common experience in computer science, soft programing and practice in artificial learning systems that randomization can offer the simplest distributed search for optimal solutions or states. Concepts from theory on communication networks may also support understanding of cellular variability. Randomization often plays a role in decentralized control and signaling in networks with local autonomy. Distribution of roles between entities generally requires communication (signaling). Analogies to collaborative behavior among higher organisms may also help to understand potential significance of variability among cells. Individuals of higher organisms typically have to prioritize between mutually exclusive activities. Randomization can be part of a system for decentralized distribution of roles to achieve synergies. Variability may also play a role in the innate immune system to obstacle intrusion.

Exchange of information can facilitate specialization where minorities in cell populations can function as sensitive sensors reporting to the others. Sensitivity, control and signaling have typically a cost in terms of energy and risk. It is, for example, a generic fact that sensitive sensors are vulnerable. The decision to take the role as special (sensitive and “expendable”) sensor on behalf of the majority should, therefore, presumably be taken by random with appropriate low probability.

The present recordings of BC3H1 cells after YTX exposure allow following single cellular events as seen in Figures 2 and 3. Individual cell variation in a clonal cell population is greater than previously recognized. Cells exhibit complex fates over time and they behave differently. Their cytoskeletons undergo extensive remodeling until they reach loss of motility before they die. Cells interact between each other and it is conceivable to believe that they might receive inductive signaling from neighboring cells. This can make correlations of lifetimes of cells in the same experimental dish and hence give different distributions derived from distinct dishes.

Precise estimates of lifetime distributions may provide information of interest in a variety of cellular studies and toxicology. The effect of a toxin may, for example, partly depend on cell cycle. Lifetime distributions may, therefore, be sensitive to phases in a synchronized cell population. However, caution must be exercised applying artificial cell synchronization. Eventual loss of cell synchrony can occur because not all the cells progress through the cell cycle at the same rate (Engelberg, 1964; Murphy et al., 1978). In fact, variability of cell-cycle kinetics is inherent from cell to cell and occurs to some extent using any synchronization method (Davis et al., 2001).

Different sets of samples of a stochastic variable (such as lifetime of a cell) will exhibit the same distribution if they were independent. However, samples of lifetimes of individual cells in separate experimental dishes tend to have different distributions. Hence, cells in the same dish do not seem to live independently. Figure 6 shows four distributions of lifetimes of BC3H1 cells after exposure to 100 nM yessotoxin. The distributions are here respectively from separate experiments where the cells are in the same Willco dish facilitating potential inter-cellular influence. The variability of the distributions may not be due to experimental errors but rather result from stochastic choices and cell–cell interactions. A statistical challenge is to test if a given observed distribution belongs to a family of distributions resulting from such an experiment. Traditional statistical assessments for equality of distributions like the Kolmogorov–Smirnov test will not apply in this situation. The treatment of these types of data may provide information on inter-cellular influence in cancer cells and help to predict their probability of metastasis. It may also help to detect change in cell populations during treatments and provide early warning for more detailed clinical investigations.

## Author Contributions

MK conceived the study and conducted the laboratory experiments, RK made the computer programming; both authors analyzed the results and wrote the manuscript.

## Conflict of Interest Statement

The authors declare that the research was conducted in the absence of any commercial or financial relationships that could be construed as a potential conflict of interest.

## Footnotes

^1^http://www.openbioimage.org/

^2^http://globe3d.sourceforge.net

^3^http://www.adacore.com/
